# Stroke in the Young

**DOI:** 10.4061/2011/271979

**Published:** 2012-01-22

**Authors:** Halvor Naess, Turgut Tatlisumak, Janika Kõrv

**Affiliations:** ^1^Department of Neurology, Haukeland University Hospital, N-5021 Bergen, Norway; ^2^University of Helsinki, Finland; ^3^University of Tartu, Estonia

Study of stroke among young adults and children has been a relatively neglected area until recently. Stroke in young adults is often considered to be rare, but this misconception is colored by the high incidence of stroke in old people. Approximately 5% of all strokes occur in people younger than 45 years of age, another 5% occur in those 45 to 50 years of age, and 1/4 occur in working aged individuals. Although stroke mortality is lower among the young, their risk to die from their stroke is almost 100 times compared to their nonstroke age counterparts, whereas the same ratio is only 4-fold in the elderly. Similarly, stroke morbidities are lower in the young, but these patients live with their neurological deficits much longer and many have to give up their work and social life. Stroke care in young people is especially demanding because it often affects work, education, and close family to a large extent and because of expected long survival. 

The study of stroke among young people is important for several reasons. The etiology of stroke is much more diverse in the young compared to old patients. This has therapeutical consequences and may affect outcome both in the short and the long term. Risk factors for stroke differ between young and old patients and may indicate separate approaches as to secondary preventive treatment. Stroke in young adults provides an opportunity to study stroke in general because of less comorbidity than in old patients. This may disclose mechanisms also relevant to older patients.

Stroke in children is rare and is associated with unique challenges. Diagnosis is often delayed because symptoms may be subtle and unspecific. Furthermore, etiology and risk factors in children with stroke differ from young adults with stroke.

Once almost neglected, now stroke in children and young adults is under intense research. Started with single-center small studies and shifted to several multicenter collaborations, scientists establish firmly many facades of stroke in children and in the young, including epidemiologic, etiologic, genetic, and prognostic features. Describing risk factor profiles has led to improved treatments. Evidence-based treatments have started to emerge, for example, in Sickle cell disease. New etiologic classifications and one international guideline for childhood stroke have appeared. Few textbooks have been published or are under preparation, and an international meeting dedicated to young stroke is planned.

This special issue is one of the numerous efforts in disseminating state-of-the-art information on the field covering a broad range of important topics as to stroke in children and young adults. It includes two case reports, two research articles, three clinical articles and 15 reviews. Seven articles deal with stroke in children, and 15 articles with stroke in young adults. Several review articles in this special issue stress the importance of extensive investigations in children with stroke including MRI to disclose the underlying etiology and risk factors. Rare causes including diabetic ketoacidosis-associated stroke, cardiac diseases, vascular abnormalities such as Moyamoya disease, but also more general causes such as dissection must be considered. A clinical study concludes that correct prophylaxis reduces the rate of recurrence in children with stroke. Other review articles disclose that more than half of the surviving children have long-term neurological sequels. A research article reports impaired cognitive development and impaired performance as to writing, reading, and arithmetic in children with stroke. A review article reports that the annual incidence of stroke in adults under 45 years ranges between 8.7 and 21 per 100,000. Several review articles show that etiology of cerebral infarction in young adults is varied. Most cerebral infarcts in the elderly are caused by conventional etiologies, for example, large-artery atherosclerotic disease, cerebral embolism (mainly atrial fibrillation), and small-vessel disease, and only 10% are caused by rare etiologies or cause remains undetermined. Conversely, one-fourth of cerebral infarcts in children and young adults are caused by unconventional etiologies and roughly one-third remains undetermined even after a complete workup. Where atrial fibrillation is a common cause of cerebral infarction in old patients, structural heart diseases including patent foramen ovale are frequent in young adults. However, a review article points out that atherosclerosis, which is a common cause of cerebral infarction in old patients, may be threatening even to the young adults. Genetic causes of stroke are more common in young adults than in old adults with stroke. Several review articles report on genetic thrombophilic disorders, genetic connective tissue diseases such as Ehlers-Danlos syndrome, and Fabry disease which is an X-linked lysosomal storage disorder. Stroke in developing countries is associated with etiologies which are uncommon in industrialized countries. Thus, a review article highlights the importance of cardioembolic stroke due to endocarditis in India. Stroke in pregnancy and the puerperium represents unique challenges. Two review articles address this rare but important topic. Previous studies have disclosed a link between migraine and cerebral infarction. However, this association is poorly understood. A review article presents several important issues which must be considered in this regard including patent foramen ovale, migraine specific drugs, and genetic components. 

Psychological adjustment is an important concern after stroke in young adults. A review article addresses this issue with particular consideration on service provision and return to work. A clinical article report that there was no difference as to ischemic stroke severity on admission and one week after stroke onset between patients younger than 50 years and patients older than 50 years. Because of long expected survival, information on long-term outcome after stroke in young adults is important. A review article provides a summary of long-term outcome both as to mortality, recurrent vascular events, and function. 

We hope that the present special issue provides important information on stroke in children and young adults and to be of help both as to treatment of our patients and in stimulating further research.

*Halvor Naess* *Halvor Naess* *Turgut Tatlisumak* *Turgut Tatlisumak* *Janika Kõrv* *Janika Kõrv*

